# Primary Immune Thrombocytopenia in Pregnancy: Pathology, Diagnosis, and Management

**DOI:** 10.1055/s-0043-1775837

**Published:** 2023-10-18

**Authors:** Jiaying Liu, Lei Zhang

**Affiliations:** 1 State Key Laboratory of Experimental Hematology, National Clinical Research Center for Blood Diseases, Haihe Laboratory of Cell Ecosystem, Institute of Hematology & Blood Diseases Hospital, Chinese Academy of Medical Sciences & Peking Union Medical College, Tianjin Key Laboratory of Gene Therapy for Blood Diseases, CAMS Key Laboratory of Gene Therapy for Blood Diseases, Tianjin, China; 2 Tianjin Institutes of Health Science, Tianjin, China

Primary immune thrombocytopenia (ITP) is an acquired autoimmune disease characterized by an isolated decrease in the peripheral blood platelet count without a clear cause. 1 The reported annual incidence rate of adult ITP is 2 to 10/100,000 and is higher in women of childbearing age. 2 3 Many factors increase the difficulty of ITP diagnosis and treatment during pregnancy, such as the decreased platelet count during normal pregnancy, accelerated platelet clearance, surgical delivery, the suspension of treatment, and immunoglobulin G antiplatelet antibodies transferred to the fetus through the placenta. 4 Early identification and correct treatment are crucial to ensuring the safety of mothers and babies.

Studies have shown that serum thrombopoietin (TPO) levels in pregnant women with ITP are higher than those in nonpregnant women with ITP, suggesting that platelet production may be impaired. 5 6 Elevated estrogen levels during pregnancy can also induce megakaryocyte maturation disorder and apoptosis. 5 7 In addition, whether the platelet membrane surface glycoprotein IIIa expressed on syncytiotrophoblasts is a potential target of autoantibodies or an inflammatory factor remains to be explored.

In the *New England Journal of Medicine* , Bussel et al reviewed the management of ITP during pregnancy. 8 Physiological thrombocytopenia is more common in pregnant women, usually starting in the first trimester and gradually decreasing during pregnancy until delivery. 9 This is associated with hemodilution and increased plasma volume. 10 ITP is the most common cause of platelet counts below 80 × 10 ^3^ per cubic millimeter and of thrombocytopenia in the first and second trimesters in healthy pregnant women. ITP manifests as isolated thrombocytopenia, and these patients have normal peripheral blood smears and show no evidence of having other hematological diagnoses, including hereditary thrombocytopenia, and those caused by drugs, infection, and other causes. A significant response to ITP therapy is also strong diagnostic evidence. 11 The diagnosis of ITP during pregnancy needs to be differentiated from other types of thrombocytopenia, especially gestational thrombocytopenia (GT). GT, by far the most common cause of thrombocytopenia during pregnancy, is associated with hemodilution from increased blood volume and increased platelet destruction as blood flows across the rough placental trophoblast surface. 9 The level of serum TPO in the peripheral blood of pregnant women with ITP is increased, which can be used as a reference index for differentiation from GT.

Although the platelet count decreases in almost all women with ITP during pregnancy, it is not common for women with platelet counts below 30 × 10 ^3^ per cubic millimeter, and the incidence of bleeding is only slightly higher than that of nonpregnant ITP patients (hazard ratio = 1.83, 95% confidence interval: 0.91–3.65). Compared with nonpregnant women with ITP, women with ITP during pregnancy are more likely to experience worsening thrombocytopenia and treatment adjustment. 12 Platelet counts usually return to prenatal levels after delivery. Most studies have found that ITP does not cause an increase in the risk of common complications of pregnancy, such as preeclampsia, preterm delivery, placental abruption, and thromboembolism.

Regarding management, this review suggested routine follow-up every 4 weeks in the first and second trimesters, then every 2 weeks thereafter, and finally weekly until delivery. The indications for treatment in the first and second trimesters of pregnancy are the same as those in nonpregnant patients with ITP (such as a platelet count < 20 × 10 ^3^ to 30 × 103 per cubic millimeter, bleeding, or invasive operation). The mode of delivery should be chosen according to obstetrical indications. Platelet counts ≥ 30 × 10 ^3^ per cubic millimeter in women with vaginal delivery, ≥ 50 × 10 ^3^ per cubic millimeter in women undergoing caesarean section, and ≥ 70 to 80 × 10 ^3^ per cubic millimeter in women with intraspinal anesthesia are recommended.

Regulatory agencies have not approved drugs for the treatment of ITP during pregnancy. Prednisone can be considered for first-line treatment, since it becomes inactivated when passing the placental barrier. The initial dose is usually 10 to 20 mg/d and is gradually adjusted to the minimum dose. When glucocorticoid therapy is not effective or when patients are ready to give birth, increasing intravenous immunoglobulin (IVIG) should be considered. The common dose is 400 mg/kg/d^^ × 5 days or 1 g/kg/d^^ × 1 ∼ 2 days.

Experts have not yet reached a consensus regarding the second-line treatment for pregnancy complicated with ITP. Rituximab may affect the immune function of newborns, so it is not recommended for the treatment of ITP during pregnancy. The safety data of immunosuppressants such as azathioprine, dapsone, and cyclosporine are mostly from other disease reports, and there is a lack of sufficient evidence-based medical evidence for pregnant ITP patients. TPO receptor agonists (TPO-RAs) are the first choice for the second-line treatment of ITP and has not been approved by regulators for use during pregnancy. A multicenter prospective study included 31 pregnant women with ITP for whom glucocorticoid and/or IVIG therapy was ineffective. They were treated with recombinant human TPO 300 U/kg/d^^ × 14 days in the second and third trimesters of pregnancy, with an overall effective rate of 74.2% and no obvious adverse reactions. 13 Another retrospective study showed that 77% of pregnant patients with refractory ITP responded to treatment with eltrombopag or romiplostim. 14 As a means of increasing the platelet count before delivery, multiple case reports support the safe and effective administration of TPO-RAs in the third trimester of pregnancy. 15 16 17 18

In summary, this review provides guidance for managing ITP in pregnant women. The selection of conventional ITP treatment drugs during pregnancy should be based on a pregnant woman's individual situation, pregnancy time, and mode of delivery.
